# *Helicobacter pylori*, Protected from Antibiotics and Stresses Inside *Candida albicans* Vacuoles, Cause Gastritis in Mice

**DOI:** 10.3390/ijms23158568

**Published:** 2022-08-02

**Authors:** Pratsanee Hiengrach, Wimonrat Panpetch, Ariya Chindamporn, Asada Leelahavanichkul

**Affiliations:** 1 Center of Excellence on Translational Research in Inflammation and Immunology (CETRII), Department of Microbiology, Chulalongkorn University, Bangkok 10330, Thailand; pratsaneeh@gmail.com (P.H.); mon-med@hotmail.com (W.P.); 2 Department of Microbiology, Faculty of Medicine, Chulalongkorn University, Bangkok 10330, Thailand; 3 Antimicrobial Resistance and Stewardship Research Unit, Faculty of Medicine, Chulalongkorn University, Bangkok 10330, Thailand; 4 Nephrology Unit, Department of Medicine, Faculty of Medicine, Chulalongkorn University, Bangkok 10330, Thailand

**Keywords:** peptic ulcer disease, gastritis, *Helicobacter pylori*, *Candida albicans*, intravacuolar *H. pylori*, bacteria-like body, stress conditions

## Abstract

Due to (i) the simultaneous presence of *Helicobacter pylori* (ulcer-induced bacteria) and *Candida albicans* in the stomach and (ii) the possibility of prokaryotic–eukaryotic endosymbiosis (intravacuolar *H. pylori* in the yeast cells) under stresses, we tested this symbiosis in vitro and in vivo. To that end, intravacuolar *H. pylori* were induced by the co-incubation of *C. albicans* with *H. pylori* under several stresses (acidic pH, non-*H. pylori*-enrichment media, and aerobic environments); the results were detectable by direct microscopy (wet mount) and real-time polymerase chain reaction (PCR). Indeed, intravacuolar *H. pylori* were predominant under all stresses, especially the lower pH level (pH 2–3). Interestingly, the *H. pylori* (an amoxicillin-sensitive strain) inside *C. albicans* were protected from the antibiotic (amoxicillin), while extracellular *H. pylori* were neutralizable, as indicated by the culture. In parallel, the oral administration of intravacuolar *H. pylori* in mice caused *H. pylori* colonization in the stomach resulting in gastritis, as indicated by gastric histopathology and tissue cytokines, similar to the administration of free *H. pylori* (extra-*Candida* bacteria). In conclusion, *Candida* protected *H. pylori* from stresses and antibiotics, and the intravacuolar *H. pylori* were able to be released from the yeast cells, causing gastric inflammation with neutrophil accumulations.

## 1. Introduction

Peptic ulcer disease and gastritis are inflammations of the mucosal and muscular layers [1,2] of the stomach and proximal duodenum that lead to several findings, including stomachache, indigestion, nausea with loss of appetite, malnutrition, positive stool occult blood, and hematemesis (in severe cases) [2,3,4]. Indeed, peptic ulcer is a worldwide health care problem, with the highest incidence in developing countries [5]; it is caused both by *Helicobacter pylori* (*H. pylori*) infection (50% of cases) and by the chronic use of non-steroidal anti-inflammatory drugs (NSAIDs) (25% of patients) [6,7]. Notably, the possible fecal-oral and oral–oral transmissions of *H. pylori* that occur among populations with poor socioeconomic conditions lead to a higher incidence of gastritis in developing countries [5]. *H. pylori* (previously named *Campylobacter pylori*) is a gram-negative spiral (helical) bacterium [8] that causes several lesions in the human gastrointestinal (GI) tract, including asymptomatic gastritis (85%), chronic gastric inflammation (15%), and gastroduodenal ulcer disease (less than 1%) that are associated with gastric carcinoma and intestinal metaplasia [9,10]. Interestingly, *H. pylori* are transmittable from person to person not only through saliva but also by fecal contamination in food and water (fecal-oral transmission), which can cause outbreaks in some communities [11]. After ingestion, *H. pylori* use tail-like flagella to move around and burrow into the epithelium (stomach and proximal duodenum) and produce urease to neutralize the gastric acidic conditions that change urea to ammonia; *H. pylori* also secrete exotoxins, such as proteins from cytotoxin-associated gene A (*CagA*) and vacuolating cytotoxin A (*VacA*), inducing gastric epithelium cell cytotoxicity and mucositis lesions [6,12,13]. Then, gastric epithelial cells are exposed to acid, which results in further cell damage and inflammatory responses from macrophages, neutrophils, lymphocytes, and plasma cells; these inflammatory responses are the main component of peptic ulcer lesions [14,15,16,17]. Recent studies have revealed that interference with the flagella functions and urease production of *H. pylori* neutralizes bacterial virulence through the inhibition of the bacterial colonization [18]. According to the conventional culture-dependent method, *H***.** *pylori* are not the only microorganism in the human stomach; fungi (especially *Candida albicans*) are also common microbiota in the human GI tract. Accordingly, the presence of *C. albicans* was identified (approximately 10^2^ CFU/mL) in the gastric contents of 70% of healthy adults [19]. Indeed, next-generation sequencing analysis, based on the presence of 18s ribosomal RNA (rRNA) and the internal transcribed spacer (ITS) gene, indicates that *Candida* spp. is an important microorganism in the human stomach [19]. Hence, interactions between *H. pylori* and *Candida* are possible.

Additionally, *C. albicans,* which are yeast-formed fungi in the phylum Ascomycota [20], are the main component of the fungal microbiota in several parts of the human body, including the oral cavity, skin, GI tract, genitourinary system, and vagina; they are categorized as opportunistic pathogens that cause invasive candidiasis (IC) in immunocompromised hosts [21]. Interestingly, *C. albicans* are able to proliferate in a highly acidic environment, such as the stomach (pH ranging from 1.5 to 3.5) [19], which possibly worsens stomach mucosal lesions. Moreover, (1, 3)-beta-D-glucan (BG), the major polysaccharide component in the yeast cell wall that is released during the growth and death of fungi [22], is one of the important pathogen-associated molecular patterns (PAMPs) that can enhance pro-inflammatory effects through macrophage and neutrophil stimulation [23,24,25,26,27,28,29,30]. Unsurprisingly, the oral administration of *C. albicans* enhances systemic inflammation and disease severity in several models through the activation of BG against innate immunity [31,32,33,34,35,36,37]. Despite the intensive study of bacterial–fungal interaction, especially *Candida* spp., in respiratory systems and catheter infections [38,39], there are few data on *Candida*–bacterial interactions in the stomach. Recently, *H. pylori*-specific genes were demonstrated inside the vacuoles of *Candida* spp. (intravacuolar *H. pylori*), and it was proposed that yeast cells were a vehicle for transmitting *H. pylori* and protecting them from stressful environments [40,41,42]. Although the synergy of *C. albicans* with *H. pylori* (outside yeast cells) [43] and other bacteria [44,45] in enhancing the severity of inflammation via inter-kingdom co-operation is demonstrated partly through the promotion of bacterial colonization by *Candida*’s epithelial adhesion property, data regarding the pathophysiology and clinical impacts of *H. pylori* inside *Candida* yeast cells are still very scarce. Here, we initiated intravacuolar *H. pylori* in vitro and tested the clinical impacts in vitro and in a mouse model.

## 2. Results

### 2.1. Induction of Intravacuolar H. pylori in Candida Yeast Cells

**Figure 1:**
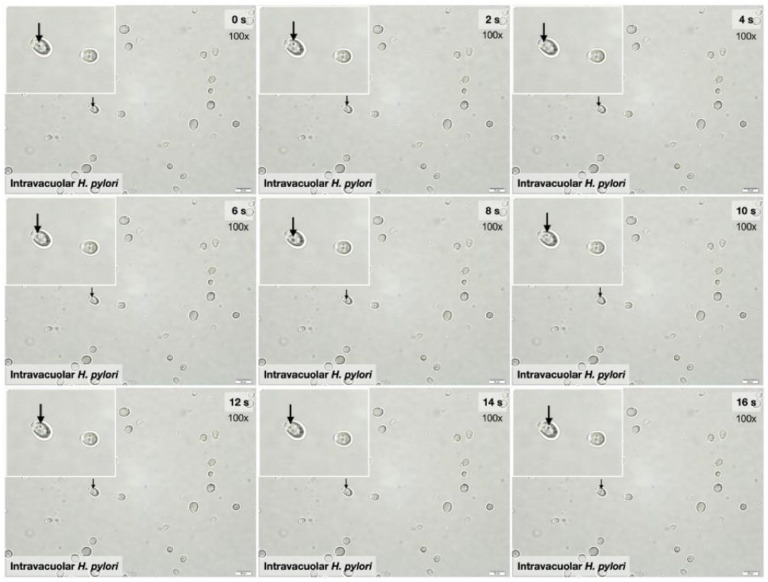
Characteristics of bacteria-like bodies (BLBs). The motile property of intravacuolar *H. pylori* in the cytosols of *Candida* yeast cells (BLBs) was demonstrated by the time-frame pictures.

**Figure 2:**
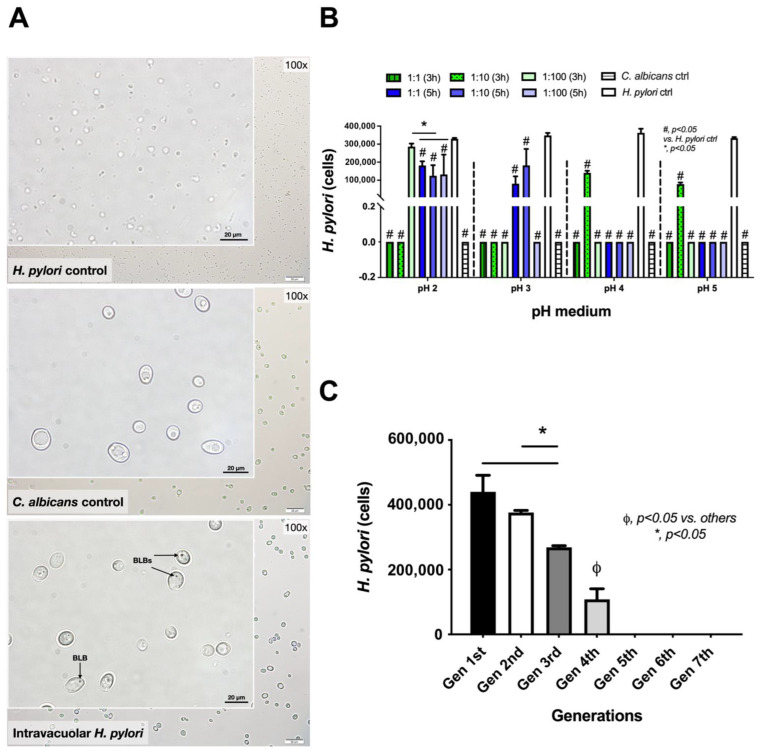
Characteristics of intravacuolar *H. pylori* inside *Candida* yeast cells under a bright-field microscope, 10 fields/slide (100× magnification), compared with the controls *H. pylori* and *C. albicans* alone (**A**). The real-time polymerase chain reaction (PCR) based on *CagA* gene expression demonstrates intravacuolar *H. pylori* in different conditions (the *H. pylori* vs. *Candida* ratio, pH media, and duration of incubation) compared with control (**B**). Intravacuolar *H. pylori* in each generation after the sub-culture of *Candida* yeast cells (**C**) (n = 9/group). Independent triplicate experiments were performed. *, *p* < 0.05; ϕ, *p* < 0.05 vs. others; BLBs, bacteria-like bodies; ctrl, control; Gen, generation of the *Candida* culture.

The endosymbiosis of *H. pylori* in yeast cells was indicated by the presence of *H. pylori* in *Candida* cytosols, referred to as “intravacuolar *H. pylori*”, as previously mentioned [45,46]. As such, *H. pylori* and *C. albicans* were co-incubated under different conditions, including the bacteria–*Candida* ratio, pH of the media, and duration of incubation); the result was the identification of *H. pylori* inside *Candida* yeast cells, as illustrated by bacteria-like bodies (BLBs) (dense black dots in a state of movement) inside the yeast cells [40,41,43,47,48,49,50], using bright-field microscopy (Figure 1). With continuous time-frame illustrations, the movements of the dense black dots could be observed (Figure 1), supporting the possible existence of BLBs inside the yeast cells. Interestingly, BLBs were detectable after the *H. pylori*–*C. albicans* co-incubation but not in the *C. albicans* culture alone (Figure 1 and Figure 2A). The higher density of *H. pylori* in BLBs may be responsible for the dense, black-colored dots observed in the cytosol of the co-incubated culture (Figure 2A lower), whereas the lower density of *Candida*’s organelles may explain the non-colored cytosol of the *Candida* culture without bacteria (Figure 2A middle). The highest abundance of intravacuolar *H. pylori* was demonstrated in a 1:100 ratio of *H. pylori* to *Candida* (1 × 10^8^ and 1 × 10^6^ CFU/mL of *H. pylori* and *C. albicans*, respectively) in Sabouraud dextrose broth (SB) media at pH 2 after 3 h incubation. There was a decrease in intravacuolar *H. pylori* in the sub-cultures starting from the 2nd generation (Figure 2B,C). At pH 2, intravacuolar *H. pylori* were detectable for all *H. pylori*: *Candida* ratios after a 5 h incubation, but only at the 1:100 ratio after a 3 h incubation (Figure 2B). At pH 3, intravacuolar *H. pylori* were detectable only after a 5 h incubation, and only for the ratios 1:1 and 1:10 (Figure 2B). Meanwhile, at pH 4 and 5, the 1:10 ratio was only the condition that could induce intravacuolar *H. pylori* (Figure 2B). Hence, the stomach (pH 1.5–3.5) is possibly the most suitable organ for the formation of intravacuolar *H. pylori* in *Candida*. This formation may depend on the abundance of *Candida* in the stomach and the gastric emptying time. The intravacuolar *H. pylori* were able to pass through the daughter yeast cells as there were similar abundances of *H. pylori* in the 1st and 2nd generations of the culture; however, *H. pylori* abundance was reduced in the 3rd and 4th generations (Figure 2C). Notably, incubation by SB (a *Candida*-enrichment media) in aerobic conditions (conditions for enhancing *Candida* growth) was the environmental stress for *H. pylori*. 

### 2.2. Release of Intravacuolar H. pylori from Candida Yeast Cells 

**Figure 3:**
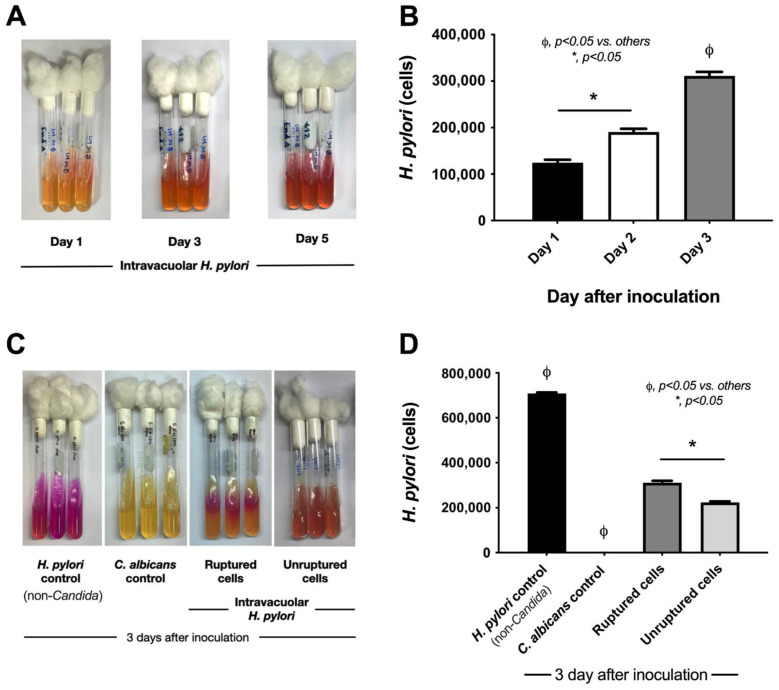
Characteristics of intravacuolar *H. pylori* released from *Candida* yeast cells. The activities of intravacuolar *H. pylori* inside *C. albicans* were demonstrated by culture on urea-based agar and quantitative real-time polymerase chain reaction (qPCR) after the culture (1, 3, and 5 days) (**A**,**B**). The results of 3 days of urea-based agar culture using sonicated samples (ruptured cells) vs. non-sonicated (unruptured cells) when compared with the *H. pylori* control or *Candida* control (**C**,**D**). Independent triplicate experiments were performed. *, *p* < 0.05; ϕ, *p* < 0.05 vs. others, as calculated by ANOVA with Tukey’s analysis.

While the endosymbiosis of *H. pylori* in *Candida* yeast cells was inducible by several stresses (high oxygen levels and a non-*H. pylori*-enrichment media) (Figure 2A–C), we further tested whether the intravacuolar *H. pylori* could be released. Without any further manipulations of the *Candida* containing intravacuolar *H. pylori*, the culture of these *Candida* yeast cells in urea-based agar demonstrated urease activity (i.e., turning the color of the media from yellow into pink) at 3 and 5 days after the culture (Figure 3A) with *H. pylori* detectable by qPCR (Figure 3B). However, the breakdown of the yeast cell wall (with sonication) facilitated the release of *H. pylori* from *Candida.* There was a higher abundance of *H. pylori* released from sonicated *Candida* yeast cells with intravacuolar *H. pylori*; bacterial abundance after a 3-day-culture in urea-based agar using the sonicated *H. pylori*-containing *Candida* cells was higher than it was in the non-sonicated samples, as determined by the color of the agar (urease test) and qPCR assay (Figure 3C,D). 

### 2.3. Intravacuolar H. pylori Was Protected from Antibiotics and Stress Conditions

**Figure 4:**
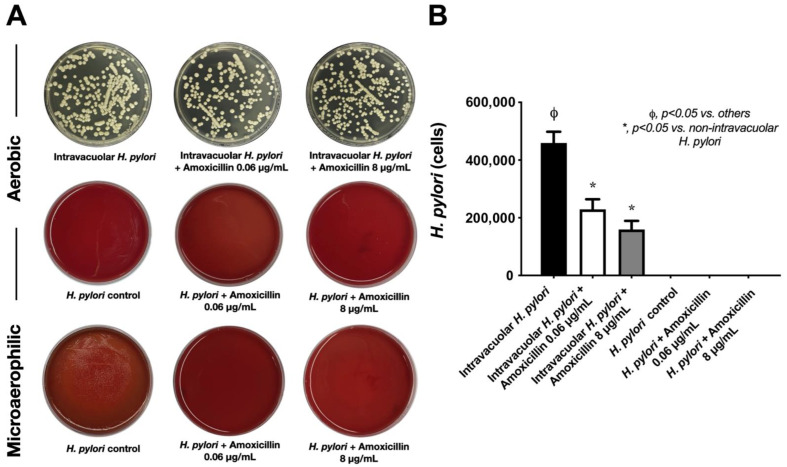
The protection of *H. pylori* by *Candida*. Representative pictures of the culture plates of second-generation intravacuolar *H. pylori* inside *C. albicans* co-incubated on SDA for 24 h with or without amoxicillin at 0.06 and 8 ug/mL (**A** upper); in addition, the culture plates of extracellular *H. pylori* in Columbia blood agar after 24 h of aerobic conditions (**A** middle) and microaerophilic conditions with amoxicillin (**A** lower) are shown. Additionally, *H. pylori* bacterial abundance based on *CagA* gene expression in these conditions is also shown (**B**). Independent triplicate experiments were performed. *, *p* < 0.05 vs. non-intravacuolar *H. pylori*; ϕ, *p* < 0.05 vs. others, as calculated by ANOVA with Tukey’s analysis.

Because a possible benefit of the *H. pylori* endosymbiosis inside *C. albicans* may be protection from antibiotics and stresses, the culture conditions of *Candida* using SB (the non-enrichment media of *H. pylori*) in aerobic conditions (a stress factor for *H. pylori*) with or without amoxicillin (a representative antibiotic) were tested against *Candida* containing *H. pylori*. Amoxicillin had no effect on *Candida* containing *H. pylori* as the antibiotic could not reduce the yeast colonies (Figure 4A upper), even though the selected strain of *H. pylori* was sensitive to aerobic conditions (*H. pylori* in Columbia blood agar in aerobic conditions) (Figure 4A middle) and to amoxicillin (no bacterial colony in Columbia blood agar in microaerophilic conditions) (Figure 4A lower). Notably, *H. pylori* were sensitive to amoxicillin in either low or high concentrations (Figure 4A lower). Likewise, the bacterial abundance of *H. pylori* based on *CagA* gene expression demonstrated the highest *H. pylori* abundance in *Candida* with intravacuolar *H. pylori* without amoxicillin, despite an aerobic environment (an environment toxic to *H. pylori*) (Figure 4B). In *Candida* containing intravacuolar *H. pylori* with amoxicillin, *H. pylori* abundance was lower than in the non-antibiotic group, but there was a non-difference between high versus low concentrations of amoxicillin (Figure 4B). Meanwhile, *H. pylori* were non-detectable in the samples of free *H. pylori* (*H. pylori* alone) under aerobic conditions and microaerophilic conditions with amoxicillin (Figure 4B). The reduced *H. pylori* abundance in amoxicillin-incubated *Candida* containing *H. pylori* may reflect the effect of amoxicillin on spontaneously released *H. pylori*. 

### 2.4. Candida Containing Intravacuolar H. pylori Caused Peptic Ulcer Disease

**Figure 5:**
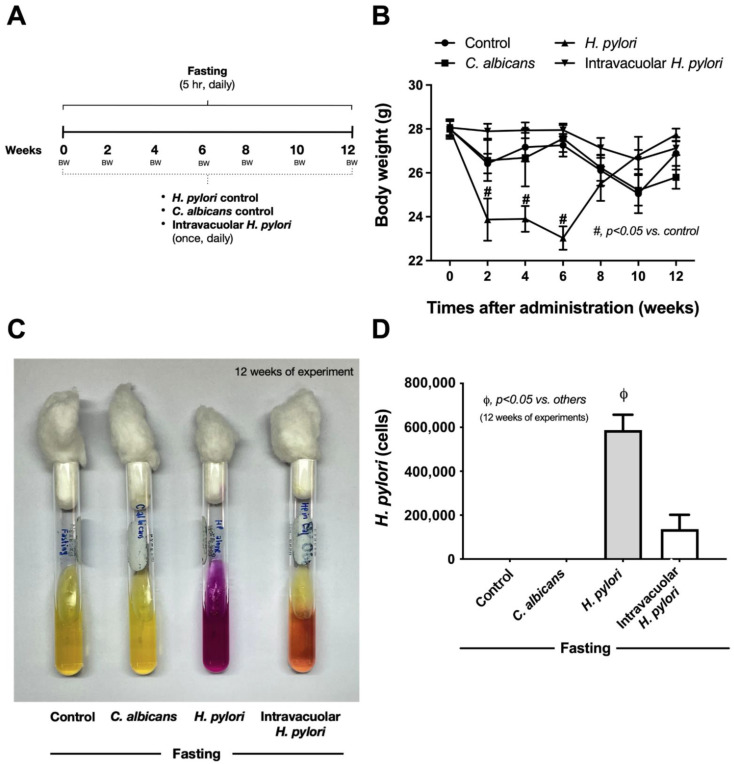
Characteristics of gastritis in mice after intravacuolar *H. pylori* administration. The schema of the experiments indicates the once-daily oral administration of 1× phosphate buffer solution (PBS) control, *C. albicans* alone, *H. pylori* alone, or *Candida* containing *H. pylori* for 12 weeks (**A**). The time-points of weight loss (%) of the mice in each group (**B**) and the abundance of *H. pylori* in the stomach at 12 weeks post-experiment, as indicated by the representative pictures of the urea-based culture (**C**) and *CagA*-gene expression (**D**) are shown (n = 6–9/group). #, *p* < 0.05 vs. control; ϕ, *p* < 0.05 vs. others, as calculated by ANOVA with Tukey’s analysis.

**Figure 6:**
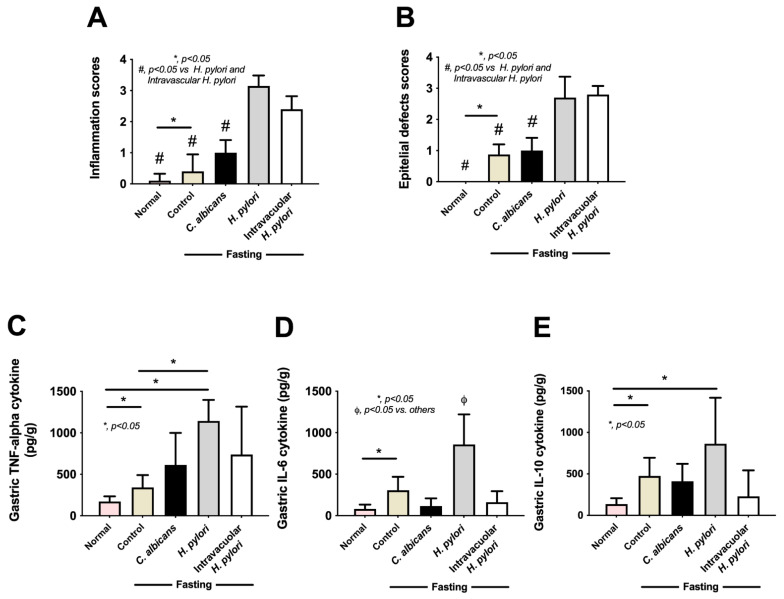
Representative pictures of histological scores; inflammatory and epithelial defects in mice stomachs (**A**,**B**) at 12 weeks after fasting and the administration of *H. pylori*, *C. albicans* (control), or *Candida* containing intravacuolar *H. pylori* are demonstrated by histological scores (**A**,**B**) and levels of gastric cytokines (IL-6, TNF-α, and IL-10) (**C**–**E**) (n = 10/group). *, *p* < 0.05; #, *p* < 0.05 vs. *H. pylori* control and intravacuolar *H. pylori* groups; ϕ, *p* < 0.05 vs. others, as calculated by ANOVA with Tukey’s analysis.

**Figure 7:**
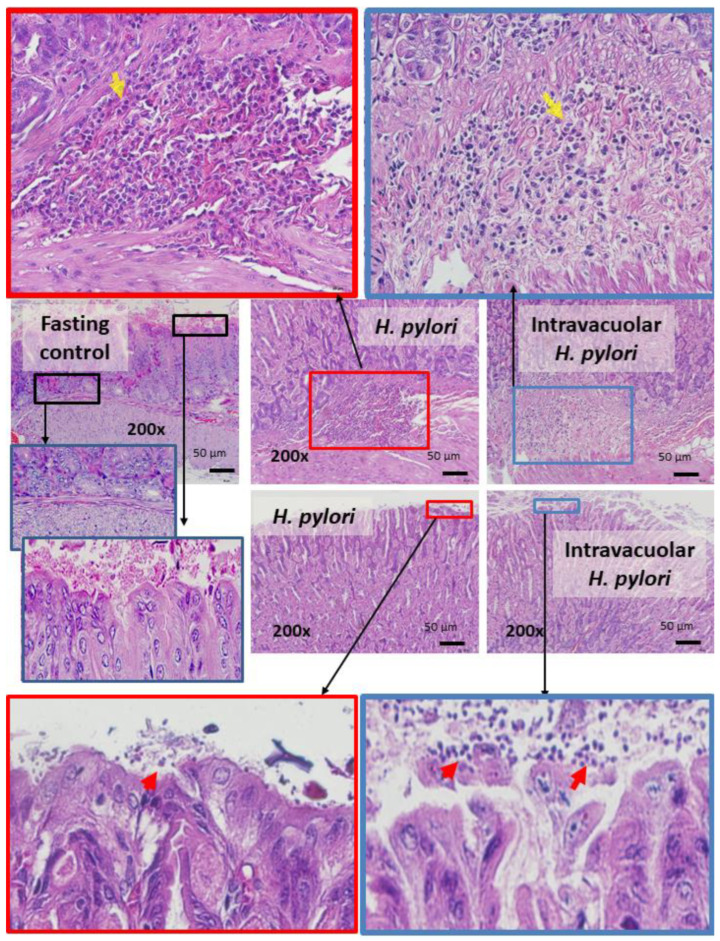
Representative pictures of the hematoxylin and eosin (H and E) staining of mouse stomachs at 12 weeks with fasting control, *H. pylori* administration, and *Candida* containing intravacuolar *H. pylori* gavage demonstrate neutrophil infiltration (yellow arrows) and mucosal mononuclear cells (red arrows) in the *H. pylori* and intravacuolar *H. pylori* groups but not in the fasting control group. The histological pictures of control mice and those who received *Candida* administration alone are not shown due to the non-difference compared to the fasting control group. The injury scores of these pictures are displayed in Figure 6A,B. A more prominent submucosal accumulation of neutrophils (polymorphonuclear cells with eosinophilic color staining) (yellow arrows) and epithelial mononuclear cells (red arrows) was observed in the *H. pylori* group than in the intravacuolar *H. pylori* mice.

Although *H. pylori* contained inside *Candida* yeast cells have been previously reported [40,41,42], the clinical importance is still not clear. To test the clinical impacts of intravacuolar *H. pylori* in *Candida* yeast cells, a gastritis mouse model was conducted using the daily oral administration of vehicle control, *H. pylori* alone, *C. albicans* alone, and *Candida* containing intravacuolar *H. pylori* for 12 weeks (Figure 5A, schema). Results showed that *H. pylori* administration induced transient weight loss, especially at 2–6 weeks of administration; then, body weight increased to a level similar to pre-administration weight (Figure 5B). Meanwhile, there was no weight change in the other groups (control, *Candida* alone, and *Candida* containing *H. pylori*) during the 12 weeks of the experiment (Figure 5B). Despite the improved body weight at 12 weeks after *H. pylori* administration, colonization by *H. pylori* was detected in the stomach, as indicated by urease activity (a pink color change in urea-based agar) and *CagA* gene expression in samples taken from the mouse stomach (Figure 5C,D). Likewise, *H. pylori* were also detected in the stomach of mice after 12 weeks of the administration of *Candida* containing intravacuolar *H. pylori* (Figure 5C,D). In parallel, *H. pylori*-administered mice demonstrated gastritis with inflammatory responses (Figure 6A–E), as indicated by the prominent histological scores based on inflammatory cell infiltration and epithelial damage (Figure 6A,B and Figure 7A,B) and increased inflammatory cytokines (IL-6 and TNF-α, but not IL-10) in the stomach (Figure 6C–E). On the other hand, the administration of *Candida* containing *H. pylori* caused less extreme histological scores and levels of tissue cytokines in the stomach than the administration of *H. pylori* alone after 12 weeks of the experiment (Figure 6A,B). There were no gastritis lesions in mice with daily fasting with 1xPBS (control) or *Candida* administration, although the levels of stomach pro-inflammatory cytokines (IL-6 and TNF-α) were higher in fasting mice with PBS or *C. albicans* than in the non-fasting control mice (normal mice) (Figure 6C–E); nevertheless, the levels observed in the fasting mice were lower than those of mice administered with either *H. pylori* or *Candida* containing *H. pylori* (Figure 6C–E). There was an abundance of inflammatory cells in the gut (submucosa and mucosa) in mice with either *H. pylori* alone or *Candida* containing *H. pylori* compared to the absence of injury in other groups (Figure 6A,B), despite a tendency toward more prominent submucosal neutrophils and epithelial mononuclear cells (Figure 7).

## 3. Discussion

Intravacuolar *H. pylori* inside *C. albicans* were protected from antibiotics (and environmental stresses) and could cause gastritis after the release of *H. pylori* from the yeast cells, illustrating the importance of natural bacteria–fungi symbiosis.

### 3.1. The Transfer of H. pylori Inside the Vacuoles of Candida Yeast Cells

*H. pylori* are vulnerable to a non-acidic micro-environment, and they cannot be isolated from food, water, or non-stomach specimens (saliva and feces). Indeed, the gastric epithelium and immune cells (macrophages and dendritic cells) are the only eukaryotic cells that host *H. pylori* (the facultative intracellular bacteria) [41]. As such, viable intracellular prokaryotes inside eukaryotes are a prokaryotic adaptation to a wide range of environmental stresses [41]. *H. Pylori* can be established not only in eukaryotic human cells but also in eukaryotic microorganisms (e.g., *Candida* spp.) as a form of “prokaryotic–eukaryotic endosymbiosis” [41,45,47] in which the endosymbiotic bacteria are localized inside the membrane-bound vacuole [40,41,47,48]. Likewise, several examples of the endosymbiosis of endobacteria inside fungi have been described [51]. For example, *Rhizopus microcarpus*, a plant pathogen fungus, provide micronutrients, such as phosphate, to *Burkholderia rhizoxinica* that stay inside the fungi [51]. Perhaps *H. pylori*’s specific ergosterol interacts with the yeast vacuole to allow the viability of “intravacuolar *H. pylori*” [41]. Because *Candida* yeast is exceptionally resistant to stressful conditions [52,53], the yeast cell can be used as an alternative host to protect *H. pylori* from stress outside the stomach, offer nourishment, and serve as a conduit for the bacterium’s spread within human populations and in the environment [41]. Indeed, the mutual cooperation between *Candida* spp. and *H. pylori* was previously mentioned; the exosymbiosis between the extracellular form of *Candida* yeast and *H. pylori* results in the synergistic production of an interkingdom biofilm [45]. However, the endosymbiosis of intravacuolar *H. pylori* inside yeast cells may be even more complex than the synergy observed in biofilm production because it indicates an evolution in the symbiosis between the structures of these organisms. 

Here, within the culture media of fungi that were too acidic for *H. pylori*, had fewer nutrients, or were non-microaerophilic, *H. pylori* could form bacteria-like bodies (BLBs) inside *C. albicans* vacuoles, as supported by a previous publication [46]. Notably, the differences between the BLBs and the non-specific components of the yeast cell’s cytosol were the movement and the density of the particles, as indicated in Figure 1 and Figure 2A. While *C. albicans* can easily adapt to acidic pH levels by secreting acids out of the cell after approximately 50 h of incubation [46], *H. pylori* are more vulnerable to extreme acidity, despite their ability to enhance pH using urease [54,55]. Moreover, fungi can also adapt to alkali environments; some pathogenic fungi secrete acids that are used for the induction of host tissue injury [56]. Interestingly, the *H. pylori*-specific gene (*CagA*) was detectable in *C. albicans* with intravacuolar *H. pylori*; this finding supports the likelihood of interaction between these two microorganisms. Due to the effectiveness of the protection, *H. pylori*’s presence inside *C. albicans* may be another mechanism explaining the persistence of *H. pylori* in the human GI tract [41,47]. Because the *CagA* gene was detectable in subsequent generations of *C. albicans*, showing vertical transmission into the daughter cells where it continued to express proteins of *H. pylori* [41], the yeast cell may be both a protective vehicle and a transmission method. Despite the use of antibiotics, including amoxicillin, together with a proton pump inhibitor for *H. pylori* eradication [57,58,59], and despite the amoxicillin susceptibility of the selected *H. pylori* in our experiments, amoxicillin could not inhibit the intravacuolar *H. pylori*, as indicated both by a culture on urea-based agar and by *CagA* expression. However, *Candida* may not protect intravacuolar *H. pylori* against the function of other antibiotics as some antibiotics may be able to diffuse inside the yeast cells. Nevertheless, the protection against amoxicillin provides proof of the concept of antibiotic protection inside yeast cells. Our data suggest that the intravacuolar *H. pylori* inside *C. albicans* were protected from antimicrobials. Hence, the development of intravacuolar *H. pylori* was very helpful for *H. pylori* in terms of resistance to treatments, extra-stomach transfers, person-to-person transmission, and environmental spread. Likewise, *H. pylori* can transform from a spiral-shaped bacillary form into a coccoid form in stress environments, similar to other Gram-negative bacteria; in this form, they are nonculturable and referred to as VBNC (viable but nonculturable) bacteria, with fewer activities than the spiral form, but they may revert to active regrowth conditions [60]. While *H. pylori*’s transformation into a coccoid form is a well-known mechanism to adapt to stresses before a regrowth that is often mentioned as an underlying mechanism for the detection of *Helicobacter* in the environment [60], intravacuolar *H. pylori* is possibly another strategy for survival in harsh environments. However, the putative infectious capability and the regrowth ability of this form of *H. pylori* are still controversial. Here, we also demonstrated that the development of intravacuolar *H. pylori* depended on pH and the abundance of *Candida*, as a higher fungal abundance at lower pH levels facilitated the intravacuolar form of *Helicobacter*. Hence, an evaluation of *Candida* abundance in the gut and the use of fungicidal drugs in the patients in whom *H. pylori* eradication has failed might be helpful. Moreover, our data also suggest that *H. pylori* inside *Candida* can be spontaneously released from the yeast cells on the 2nd and 3rd days of the symbiosis with biological activity intact (positive urease test). The prospect of additional studies on this topic is interesting. 

### 3.2. Intravacuolar H. pylori Inside C. albicans as a Cause of Gastritis

**Figure 8:**
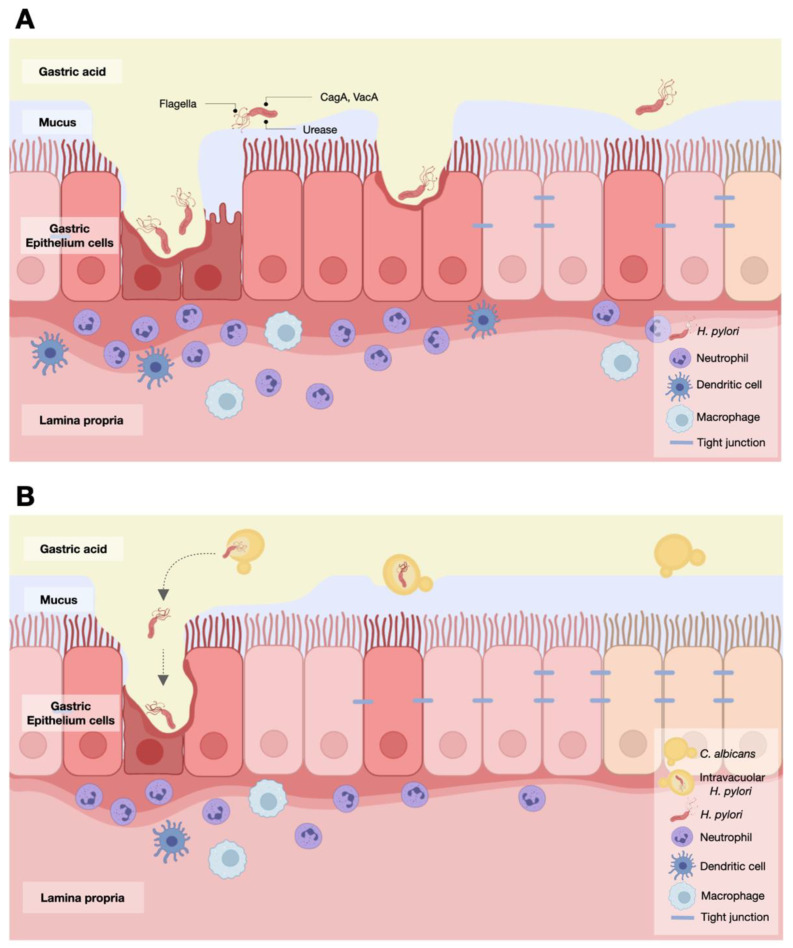
The proposed working hypothesis demonstrates the role of *H. pylori* with and without *Candida* in gastritis. Without *Candida*, *H. pylori* activate immune cells and enhance gastric inflammation (**A**). With *Candida*, the intravacuolar *H. pylori* are released from the yeast cells and induce gastric inflammation (**B**). This picture was created by BioRender (https://app.biorender.com/, accessed on 5 March 2022).

Although the administration of *C. albicans* alone does not cause gastritis, the worsening of *H. pylori*-induced gastritis by *Candida* through synergistically elevated gastric inflammation has been previously described [13,43,45]. Likewise, *Candida* alone did not induce stomach injury in our study, but gastritis was demonstrated in the mice orally administered both with intravacuolar *H. pylori* (the bacteria inside *Candida*) and with *H. pylori* alone, as indicated by inflammatory lesions, *CagA* expression, a urea-based culture, and tissue cytokine levels in the stomach. Notably, the spontaneous bodyweight regains observed in the *H. pylori* mouse model [61,62] and the absence of weight gain in mice during the fasting experiments have been previously mentioned [63]. Our data suggest that *Candida* yeast is a reservoir of *H. pylori* and that the bacteria can be released outside the yeast cells and regrow in the stomach epithelium. Although the mechanisms of *H. pylori*’s release from the yeast cells are still unknown, vesicular release has been identified as a new fungal secretory pathway that might play a role here [64]. As such, some yeast fungi, including *C. albicans*, *Cryptococcus neoformans*, and *Saccharomyces cerevisiae*, can produce vesicles containing RNA or microRNA (miRNA) (the non-coding RNA sequence with gene regulatory effects) [65,66,67] that can alter the gene expression of target cells [68]. In addition, the fungal cell wall has a dynamic structure with flexible viscoelastic qualities that allow the release of vesicles [69], as well as fungal–host communication with some specific environmental factors (pH, temperature, presence of serum, and lack of some amino acids) that trigger the production and release of vesicles [70]. Thus, these data suggest that the large vesicles of intravacuolar *H. pylori* possibly pass through the cell wall of *C. albicans* yeast cells. With the release of intravacuolar *H. pylori*, *Helicobacter* bacteria can induce gastritis through decreases in mucus and mucosal blood flow, prostaglandin synthesis, cell healing processes, and gastric emptying time, similar to regular *H. pylori* [7,8,12,71]. Notably, the abundance of *H. pylori* in the stomach of *H. pylori*-administered mice was higher than in mice with *Candida* containing intravacuolar *H. pylori* (Figure 5C,D), suggesting that some intravacuolar *Helicobacter* could not be released from the *Candida* yeast cells. In contrast, *Candida* administration alone did not cause ulcers. The severity of the gastritis caused by *H. pylori* alone versus that caused by *H. pylori* inside *Candida* was not different, indicating a lack of synergy between *H. pylori* and the *Candida* hosting it; this finding differs from previous reports of enhanced pro-inflammation with the co-presence of *H. pylori* and *Candida* [13,43,45]. Perhaps the *Candida* yeasts that host *H. pylori* lose their pro-inflammatory properties after the release of *H. pylori*. We hypothesize that the release of *H. pylori* from *C. albicans* vesicles enhances the pro-inflammatory response of enterocytes (Figure 8A,B) that activate the gastric inflammation of mucosa through Toll-like receptors, especially TLR4 and TLR2, with several adapter molecules (MyD88, IRAK1, IRAK4, and NF-κB) [72]. Hence, our data suggest that *Candida* yeast cells could be the eukaryotic hosts that protect the prokaryotic bacteria from antibiotics and stressful environments and that the hosted bacteria are ready to be spontaneously released or let out by the breakdown of the yeast cells and/or other mechanisms. More mechanistic studies on this topic would be interesting.

For the clinical translation, the spontaneous development of intravacuolar *H. pylori* inside *Candida* yeast cells may depend on the ratio of the abundances of *Candida* and *H. pylori* in each patient; such development would possibly induce resistance to the standard treatment. Currently, the causes of treatment failure for *H. pylori* focus on (i) increased antibiotic resistance [73] through the genes that affect the alteration of proton pump inhibitor pharmacokinetics and/or the host cytochrome CYP2C19 and (ii) host factors, such as poor adherence, insufficient duration of therapy, and smoking [74,75,76]. On the other hand, our data support another underlying mechanism of treatment failure through an increased abundance of fungi in the stomach. Indeed, advanced age and the consumption of proton pump inhibitors correlate with fungal colonization in the stomach, which may be associated with *H. pylori*-positive status and dyspepsia [13]. Hence, reducing the abundance of *Candida* (anti-fungal treatments) together with *H. pylori* treatment (triple therapy) may improve the outcome of treatment and reduce the recurrent rate of gastritis, especially in cases of treatment failure. Unfortunately, the correlation between *Candida* abundance and the treatment resistance of *H. pylori* is still unknown. In contrast, an interkingdom correlation between *Candida* and *H. pylori* may be clinically possible because the generation of intravacuolar *H. pylori* was easily induced by the co-incubation of both organisms in a fungal culture media, and the intravacuolar *H. pylori* could pass through the daughter yeast cells, protected from antibiotics, and possibly caused resistance to the standard treatment. More studies are warranted. 

## 4. Materials and Methods

### 4.1. Inducing Intravacuolar H. pylori in Candida Yeast Cells

*H. pylori* ATCC 43504 (Manassas, VA, USA) was cultured on Columbia agar (HiMedia, Mumbai, India) supplemented with 5% sheep blood and 7% horse serum (Invitrogen, Waltham, MA, USA) under microaerophilic conditions (5% O_2_, 15% CO_2_, and 80% N_2_) at 37 °C for 3 days. In parallel, *C. albicans* ATCC 90028 (Microbiologics, Saint Cloud, MN, USA), a fluconazole-susceptible strain (minimal inhibitory concentration 0.25–1 μL/mL), was cultured on Sabouraud dextrose agar (SDA) (Oxoid, Basingstoke, Hampshire, UK) in aerophilic conditions at 37 °C for 2 days before using. Then, *H. pylori* at 1 × 10^6^, 1 × 10^7^, and 1 × 10^8^ CFU/mL and 1 × 10^6^ CFU/mL of *C. albicans* were co-incubated with shaking in Sabouraud dextrose broth (SB) (Oxoid) at pH 2, pH 3, pH 4, and pH 5 for 3 h and 5 h. Then, the bacteria-like bodies (BLBs) inside *Candida* yeast cells were observed under a bright-field microscope (100× magnification), and 100 μL of these co-incubated microorganisms were plated on SDA with chloramphenicol (Oxoid) and incubated at 37 °C for 3 days for the *C. albicans* growth. 

### 4.2. Determination of Intravacuolar H. pylori Inside the Candida Yeast Cells

*Candida* yeast cells in each experimental group were sub-cultured to produce other generations in SDA with chloramphenicol, and a specific *H. pylori* gene inside each *Candida* generation was examined, following a previous protocol [77]. In brief, 1 × 10^6^ CFU/mL of *Candida* yeast cells were sonicated with a Beadbeater (OK 74005) (BioSpec Products Inc., Bartlesville, OK, USA) for 15 s to destroy the fungal cell wall, and phenol-chloroform was used for the extraction of DNA from the samples. Real-time polymerase chain reaction (PCR) assays were run on a QuantStudio 5 Real-Time PCR (qPCR) system (Thermo Fisher Scientific, Waltham, MA, USA) using a *CagA* gene primer to identify *H. pylori* inside *C. albicans* with the following primer sequence: forward, 5′-AGT AAG GAG AAA CAA TGA-3′ and reverse, 5′-AAT AAG CCT TAG AGT CTT TTT GGA AAT C-3′ [77]. The *H. pylori* gene expression was calculated and translated into *H. pylori* cell-abundance by the gene reference from the National Center for Biotechnology Information (NCBI; NIH, Bethesda, MD, USA). To demonstrate the release of intravacuolar *H. pylori* from the *Candida* yeast cells, the second generation of *Candida* yeast cells from the sub-culture passages was incubated in aerophilic conditions at 37 °C for 5 days using a urea agar-based culture media (400,086) (HiMedia, Mumbai, India) to demonstrate the activities of intravacuolar *H. pylori*. To see whether damaged yeast cell walls can release more *H. pylori* than non-damaged yeast cells, the *CagA* gene in the second generation of *Candida* yeast cells with or without sonication was extracted by a Beadbeater machine for whole DNA, and the presence of the *CagA* gene was determined by a qPCR system (Thermo Fisher Scientific).

### 4.3. Antimicrobial Incubation and Stresses against Intravacuolar H. pylori

To explore the possible benefits of the endosymbiotic *H. pylori* inside *C. albicans* in terms of protection from antibiotics and environmental stresses, amoxicillin, and an aerobic (high-oxygen) condition, which is a stress factor for microaerophilic *H. pylori* (i.e., it has the ability to grow in 5–15% oxygen), were tested. As such, the second generation of *Candida* yeast cells was cultured into SB with or without amoxicillin (0.06 and 8 ug/mL) (Tianjin TEDA Steyuan Pharm Co., Ltd., Shijiazhuang, Hebei, China) in aerophilic conditions (21% oxygen) at 37 °C overnight. Then, 100 μL of each sample was plated onto SDA and incubated in aerophilic conditions at 37 °C overnight. After that, the whole DNA was extracted to identify *CagA* gene expression in each experimental group using a qPCR system (Thermo Fisher Scientific).

### 4.4. Animal and Peptic Ulcer Model

The animal study (SST 018/2562) was approved by the Institutional Animal Care and Use Committee of Chulalongkorn University’s Faculty of Medicine following the animal care and use procedure of the National Institutes of Health (NIH). Male 8-week-old C57BL/6 mice weighing 20–25 g was purchased from Nomura Siam International, Pathumwan, Bangkok, Thailand. The mice were housed in a temperature-controlled environment (24 ± 2 °C), with 50% relative humidity and a 12 h light–dark cycle (light from 7:00 a.m. to 7:00 p.m.). All mice received food and water ad libitum. Animal procedures were performed in adherence with U.S. National Institutes of Health guidelines and followed the 8th Edition of the Guide for Care and Use of Experimental Animals, published by the National Research Council of the National Academies (2011; available at https://grants.nih.gov/grants/olaw/guide-for-the-care-and-use-of-laboratory animals.pdf, accessed on 12 November 2021), as well as the Animal Research: Reporting of In Vivo Experiments (ARRIVE) guidelines. 

A peptic ulcer model of *H. pylori* infection was modified from a previous study [78]. Briefly, *H. pylori* at 3.7 × 10^6^ CFU/mL, *C. albicans* at 1 × 10^8^ CFU/mL, or *C. albicans* with intravacuolar *H. pylori* at 1 × 10^8^ CFU/mL (with approximately 3.7 × 10^6^ CFU/mL of intravacuolar *H. pylori* inside the *C. albicans*) in 1 mL of 1x phosphate buffer solution (PBS) was once daily orally administered using a stainless-steel feeding tube (18-gauge size and 1.5 inches in length, with a rounded tip attached to a 1 mL syringe). Notably, all groups of mice fasted for 5 h before microorganismal administration. All mice were observed and sacrificed 12 weeks after the beginning of the experiment. Then, the stomach was divided longitudinally through the greater and lesser curvature into several parts, washed with 1xPBS, weighed, and used for determining inflammatory responses and fungal–bacterial interactions.

### 4.5. Mouse Gastric Analysis 

Mouse stomachs were divided into 4 small pieces for performing (i) a *H. pylori* culture, (ii) an analysis of *CagA* gene expression, (iii) the measurement of gastric cytokine levels, and (iv) a histopathology analysis (fixed in 10% formaldehyde). To determine *H. pylori* abundance in mouse stomachs, the gastric tissues (0.01 g per sample) were cultured on urea-based agar and incubated at 37 °C overnight before colony enumeration. In addition, the *CagA* gene was identified by qPCR (Thermo Fisher Scientific) from gastric tissues (1 mL PBS per g tissue) that were sonicated with the setting of pulse-on for 20 s and pulse-off for 5 s in 30 min on ice using the Sonics Vibra Cell machine (VCX 750) (Sonics & Materials Inc., Newtown, CT, USA) until a homogeneous solution was formed. The supernatant, after the centrifugation, was used for the detection of gastric cytokines (TNF-α, IL-6, and IL-10) and *CagA* expression by ELISA assays (Invitrogen, Waltham, MA, USA) [79,80] and PCR, respectively. For histology, the stomach tissues were rinsed with PBS, fixed in 10% (weight by volume (W/V)) formaldehyde, embedded in paraffin, and stained with hematoxylin and eosin (H and E) color in 5.0 mm thickness sections. The histological analyses of 200× magnification slides were performed by 2 observers who were blinded to the experiments; the analyses used semi-quantitative scores based on inflammatory cell infiltration (macrophages and neutrophils), mucosal damage, and ulceration on a scale of 0 to 4 as modified from a previous publication [81]. 

### 4.6. Statistical Analysis

All data were analyzed by Statistical Package for Social Sciences software (SPSS 22.0, SPSS Inc., Chicago, IL, USA) and Graph Pad Prism version 7.0 software (La Jolla, CA, USA). Results were presented as mean ± standard error (SE). The differences between multiple groups were examined for statistical significance by one-way analysis of variance (ANOVA) with Tukey’s analysis. The survival analysis and time-point data were determined by the log-rank test and repeated measures ANOVA, respectively. A *p*-value < 0.05 was considered statistically significant.

## 5. Conclusions

In conclusion, intravacuolar *H. pylori* benefit from increased transmissibility, and the fungal host protects the bacteria from stressful micro-environments, including antibiotics. Our mouse model provides evidence that intravacuolar *H. pylori* were able to induce gastric infection, inflammatory cell infiltration, and tissue damage. Future studies on the role of intravacuolar *H. pylori* in yeast cells are needed to extend our understanding of intravacuolar *H. pylori* colonization in humans in order to optimize and individualize health strategies.

## Data Availability

Data are contained within the article.
